# Preoperative Prediction Nomogram Based on Integrated Profiling for Glioblastoma Multiforme in Glioma Patients

**DOI:** 10.3389/fonc.2020.01750

**Published:** 2020-10-21

**Authors:** Wei Wu, Zhong Deng, Wahafu Alafate, Yichang Wang, Jianyang Xiang, Lizhe Zhu, Bolin Li, Maode Wang, Jia Wang

**Affiliations:** ^1^Department of Neurosurgery, The First Affiliated Hospital of Xi'an Jiaotong University, Xi'an, China; ^2^Center of Brain Science, The First Affiliated Hospital of Xi'an Jiaotong University, Xi'an, China; ^3^Department of Breast Surgery, The First Affiliated Hospital of Xi'an Jiaotong University, Xi'an, China; ^4^Department of Cardiology, The First Affiliated Hospital of Xi'an Jiaotong University, Xi'an, China

**Keywords:** preoperative prediction, GBM, nomogram, integrated profiling, diagnosis

## Abstract

**Introduction:** Traditional classification that divided gliomas into glioblastoma multiformes (GBM) and lower grade gliomas (LGG) based on pathological morphology has been challenged over the past decade by improvements in molecular stratification, however, the reproducibility and diagnostic accuracy of glioma classification still remains poor. This study aimed to establish and validate a novel nomogram for the preoperative diagnosis of GBM by using integrated data combined with feasible baseline characteristics and preoperative tests.

**Material and method:** The models were established in a primary cohort that included 259 glioma patients who had undergone surgical resection and were pathologically diagnosed from March 2014 to May 2016 in the First Affiliated Hospital of Xi'an Jiaotong University. The preoperative data were used to construct three models by the best subset regression, the forward stepwise regression, and the least absolute shrinkage and selection operator, and to furthermore establish the nomogram among those models. The assessment of nomogram was carried out by the discrimination and calibration in internal cohorts and external cohorts.

**Results and discussion:** Out of all three models, model 2 contained eight clinical-related variables, which exhibited the minimum Akaike Information Criterion (173.71) and maximum concordance index (0.894). Compared with the other two models, the integrated discrimination index for model 2 was significantly improved, indicating that the nomogram obtained from model 2 was the most appropriate model. Likewise, the nomogram showed great calibration and significant clinical benefit according to calibration curves and the decision curve analysis.

**Conclusion:** In conclusion, our study showed a novel preoperative model that incorporated clinically relevant variables and imaging features with laboratory data that could be used for preoperative prediction in glioma patients, thus providing more reliable evidence for surgical decision-making.

## Introduction

Gliomas are the most common type of neuroepithelial neoplasm in the central nervous system (CNS) and account for ~3% of all systemic malignant tumors (1–3). Gliomas are traditionally divided into four grades according to the World Health Organization (WHO) classification and The Cancer Genome Atlas (TCGA) categorization, depending on the pathological signatures and specific molecular biomarkers (4, 5). The WHO grade II and grade III are defined as lower grade gliomas (LGG) while the WHO grade IV is identified as glioblastoma multiforme (GBM) which exhibits more aggressive and invasive features (6). Accumulating evidence showed that overall survival for GBM patients was remarkably prolonged after receiving a comprehensive clinical strategy including maximal surgical resection, radio treatment, and chemotherapy (7–9). Therefore, the precision of the preoperative classification of gliomas is crucial in deciding the pertinent operative strategy and for providing adequate information to the patient (10). However, there are few methods available for pre-surgical prediction of pathological grade in glioma patients. Recent studies recommended using an integrated classification system that combines histologic classification and genetic information, such as 1p/19q chromosomal co-deletion, IDH1 mutation, EGFR amplification, and BRAF mutation (4, 5). One obvious weakness for the current histopathological grading or molecular diagnose of gliomas is that these examinations can only be obtained and confirmed after surgical resection. This delayed process resulted in an ambivalent situation in which the clinicians cannot achieve sufficient evidence to help them formulate an operation strategy. Therefore, accurate pre-surgical prediction of the prognostic classification becomes an urgent need to improve the outcomes for glioma patients.

Recent studies suggested that the non-specific immune inflammatory response contributes to different signatures associated with glioma pathogenesis, overall survival, or response to treatment (11–15). The count of immune cells from preoperative blood routines were significantly related to the WHO classification and prognosis in glioma patients (16, 17). Immune responses in GBM is characterized by low peripheral lymphocyte counts, impaired mitogen-induced responses of peripheral mononuclear cells, and accumulation of CD8^+^ suppressor T cells and CD4^+^CD25^+^FoxP3^+^Treg cells, which have been reported to play a crucial role in cancer immune surveillance and defense by inducing cytotoxic cell death and inhibiting glioma cell proliferation and migration of glioma (18–20). Monocytes specifically were recruited from peripheral blood and associated with microglia to form the tumor-associated macrophage (TAMs), which proved to be essential for tumor microenvironment regulation and promoted tumorigenesis and metastasis via secretion of inflammatory factors, thus inducing activation of peripheral blood inflammatory cells (PBICs) in gliomas (21, 22). To further evaluate the clinical relevance between immune response and prognosis of tumor patients, the systemic inflammation response index (SIRI) based on peripheral neutrophil (N), monocyte (M), and lymphocyte (L) counts was used as a survival-related predictor in multiple solid tumors, including gastric cancer, hepatocellular carcinoma, and pancreatic cancer (11, 23, 24). Despite these findings, the functional role of SIRI in gliomas still remains unclear.

MRI could provide primary investigations of the subtype and malignancy of brain tumors, thus affording a potential presumptive diagnosis for further therapeutic regimens. Previous MRI-based radio genomics studies presented a nomogram for preoperative prediction molecular subgrouping for patients with medulloblastoma by using MRI features of tumors. Henker et al. also demonstrated that preoperatively measured necrosis volume and necrosis-tumor ratio was the most crucial radiological features of GBM with a strong influence on OS. Therefore, MRI features of gliomas could be rational for preoperational grading of gliomas (25, 26).

A single predictive index provides insufficient information on gliomas. Meanwhile, accumulating laboratory examinations or radiography was relevant with GBM progression (27, 28). However, the predictive value of a combination with SIRI and MRI in the preoperative diagnosis of GBM still remains unclear. Furthermore, the traditional statistical strategy, called “data snooping,” only adopted the variables which were significant on univariate analysis to establish the final prediction models, which led to model overfitting and showed poor results (29). There are some advanced statistical methodologies to minimize this limitation, such as the best subsets regression (BSR), the forward stepwise regression (FSR), and the least absolute shrinkage and selection operator (LASSO) (30–33). Therefore, our studies aimed to establish an effective and non-invasive nomogram for preoperative diagnosis and grading methods of gliomas by using feasible baseline measurements and preoperative tests combined with SIRI and MRI, as well as adopting advanced statistical analysis.

## Method

### Patients

The flow diagram for this study was described in Supplementary Figure 1. Clinical information of all glioma patients was consecutively enrolled and this study was approved by the Ethics Committee of the First Affiliated Hospital of Xi'an Jiaotong University. This was a retrospective study, for which formal consent was not required. All included patients were carefully screened for the following inclusion criteria: (a) pathologically diagnosed grade II–IV glioma based on the WHO classification of CNS tumors (2016) (4) which was performed by two independent pathologists, (b) no history of craniotomy or stereotactic biopsy, (c) available brain MRI and blood routines in the pre-surgery period and MRI features that were measured by two independent radiologists, (d) complete clinical characteristics, and (e) no disease causing elevated or decreased PBIC. Finally, a total of 365 patients with gliomas who underwent surgical resection at the Department of Neurosurgery, the First Affiliated Hospital of Xi'an Jiaotong University from January 2014 to November 2016 were enrolled in this retrospective study.

All patients were arranged in chronological order. The top 70% of patients were assigned as the primary cohort and the bottom 30% of patients were identified as an internal validation cohort. Validation of the nomogram was also evaluated in an independent external validation cohort which included 159 patients from June 2018 to April 2019 who underwent craniotomy in our hospital.

### Clinical Characteristics

The clinical information, including sex, age, height, body weight, preoperative Karnofsky performance status (pKPS), tumor grade, the preoperative epilepsy occurrence (pEO), and preoperative blood routine tests including neutrophil, monocyte, and lymphocyte counts, were obtained from medical records. Features of MRI, comprising of tumor volume, location, multifocality, annular enhancement, necrosis volume, and peritumoral edema volume (PTE), were also involved in our analysis.

Body mass index (BMI) was defined as: BMI = height/body weight^2^. The threshold value for pKPS was <70 and ≥70 on the basis of WHO's standards (4). The SIRI was defined as: SIRI = N^*^M/L (22) and the PTE, tumor volume, and necrosis volume were calculated based on formulas described in the Supplementary Materials and Methods (26).

### Variables Selection

In order to avoid over-fitting or under-fitting of the model, three advanced statistical methods, the BSR, the LASSO, and the FSR, were adopted to select variables in the primary cohort. The criterion of variable selection for the BSR and the FSR was determined by the Bayesian information criterion (BIC) (34).

### Model Establishment

GBM-related predictive models were established in the primary cohort based on the selected variables by adopting binary logistic regression. Eventually, we developed three models as described below: (a) model 1 consisted of seven variables based on the BSR, (b) model 2 included SIRI besides the above seven variables according to the LASSO, and (c) model 3 also consisted of seven variables except for pEO according to the FSR.

The final model was determined by the Akaike Information Criterion (AIC) (35), the ROC curves, the Harrell concordance index (C-index), and the integrated discrimination index (IDI) (36, 37).

Similarly, these methods were also used to assess the performance of prediction models. Herein, the discrimination of the model was evaluated by the C-index among three cohorts, and the IDI was used to estimate whether the model's predictive ability could become better by adding one variable in model 1 or model 3. Conclusively, the nomogram was derived from the final model.

### Apparent Performance of the Nomogram

The performance of the nomogram was also demonstrated by the calibration curve among three cohorts, except for the ability of discrimination. Meanwhile, the appropriateness of the current predictors involved in nomogram was tested. As a result, the Hosmer–Lemeshow test, the studentized residuals, the variance inflation factor (VIF), the Cook's distance, the hat value, and the Box-Tidwell test were performed to evaluate model fitting, outliers, collinearity, influential observations, high leverage cases of data, and the linear relationship between continuous independent variables and the logit transformation value of the dependent variable, respectively.

### Clinical Usage

The decision curve analysis (DCA) was performed to assess the clinical usage of nomogram and a net benefit for diverse prediction models at different threshold probabilities by adding the benefits and minimizing the harms (38).

### Statistical Analysis

All the statistical analyses were performed with the SPSS software (version 22.0, SPSS Inc., Chicago, IL) and R software (version 3.2.6; http://www.r-project.org). The packages in R which we used in this study were shown as follows: “glment,” “rms,” “proc,” “PredictABEL,” “rmda,” “car,” “leaps,” and “regplot.” Statistical significance levels were determined by two-sided tests and *P* < 0.05 was defined as statistically significant. The Mann–Whitney *U*-test was used for comparing two groups of continuous variables analysis, the Kruskal–Wallis *H*-test was used for three groups' continuous variables analysis, and the χ2 test for categorical variables analysis. The BSR, the LASSO, and the FSR were used to select variables. The binary logistic regression was performed for model construction.

## Results

### Clinical Characteristics

The baseline clinical and pathological characteristics of the three cohorts were presented in Table 1. The baseline characters showed gratifying similarity in the prevalence of GBM among the three cohorts (*P* = 0.682). The proportion of GBM was 55.2, 54.7, and 50.9% among the three cohorts, respectively. Also, the baseline clinical parameters, laboratory tests, and MRI factors showed no significant differences and were comparable among the three cohorts. Additionally, the clinical information after subgroup stratification based on pathological grade in all cohorts also showed none statistical significance (Supplementary Table 1). Altogether, all selected parameters in this study showed homogeneity and comparability among all cohorts, indicating that the source and collection for the clinical data were reliable and guaranteed with high-quality.

**Table 1:** Clinical characteristics of patients in the primary and validation cohorts.

**Characteristic**	**Primary cohort**	**Internal validation cohort**	**External validation cohort**	***P***
Sex				0.943
Male	139 (53.7%)	58 (54.7%)	88 (55.3%)	
Female	120 (46.3%)	48 (45.3%)	71 (44.7%)	
Age, median (IQR), years	49.00 (36.50–58.50)	49.50 (38.00–57.50)	50.00 (38.00–58.00)	0.915
BMI, median (IQR), kg/m^2^	22.20 (20.70–23.53)	22.17 (20.75–23.88)	22.49 (21.17–23.13)	0.148
pKPS				0.691
<70	78 (30.1%)	29 (27.4%)	42 (26.4%)	
≥70	181 (69.9%)	77 (72.6%)	117 (73.6%)	
Tumor grade				0.682
LGG	116 (44.8%)	48 (45.3%)	78 (49.1%)	
GBM	143 (55.2%)	58 (54.7%)	81 (50.9%)	
pEO				0.387
Yes	88 (33.9%)	36 (34.0%)	64 (40.3%)	
No	171 (66.1%)	70 (66.0%)	95 (59.7%)	
SIRI	1.40 (0.79–2.55)	1.35 (0.70–1.94)	1.37 (0.64–2.06)	0.194
Tumor volume, median (IQR), cm^3^	32.38 (15.76–50.71)	31.44 (13.43–49.18)	33.02 (15.99–50.18)	0.368
Tumor location				0.891
Supratentorial	133 (51.4%)	57 (53.8%)	81 (50.9%)	
Infratentorial	126 (48.6%)	49 (46.2%)	78 (49.9%)	
Tumor multifocality				0.419
Yes	50 (19.3%)	26 (24.5%)	29 (18.2%)	
No	209 (80.7%)	80 (75.5%)	130 (81.8%)	
Annular enhancement				0.393
Yes	175 (67.6%)	69 (65.1%)	97 (61.1%)	
No	84 (32.4%)	37 (34.9%)	62 (38.9%)	
Tumor necrosis volume, median (IQR), cm^3^	16.72 (9.56–23.25)	15.19 (9.11–22.89)	16.28 (8.98–22.96)	0.274
PTE, median (IQR), cm^3^	41.52 (20.69–63.76)	40.38 (20.08–62.69)	41.17 (20.91–63.33)	0.597

*IQR, interquartile range; pKPS, preoperative Karnofsky performance status; pEO, preoperative epilepsy occurrence; LGG, lower grade glioma; GBM, glioblastoma multiforme; SIRI, systemic inflammation response index; PTE, peritumoral edema; P is obtained from the Kruskal–Wallis H-test and the χ2-test*.

### Variables Selection Using the BSR

**Figure 1:**
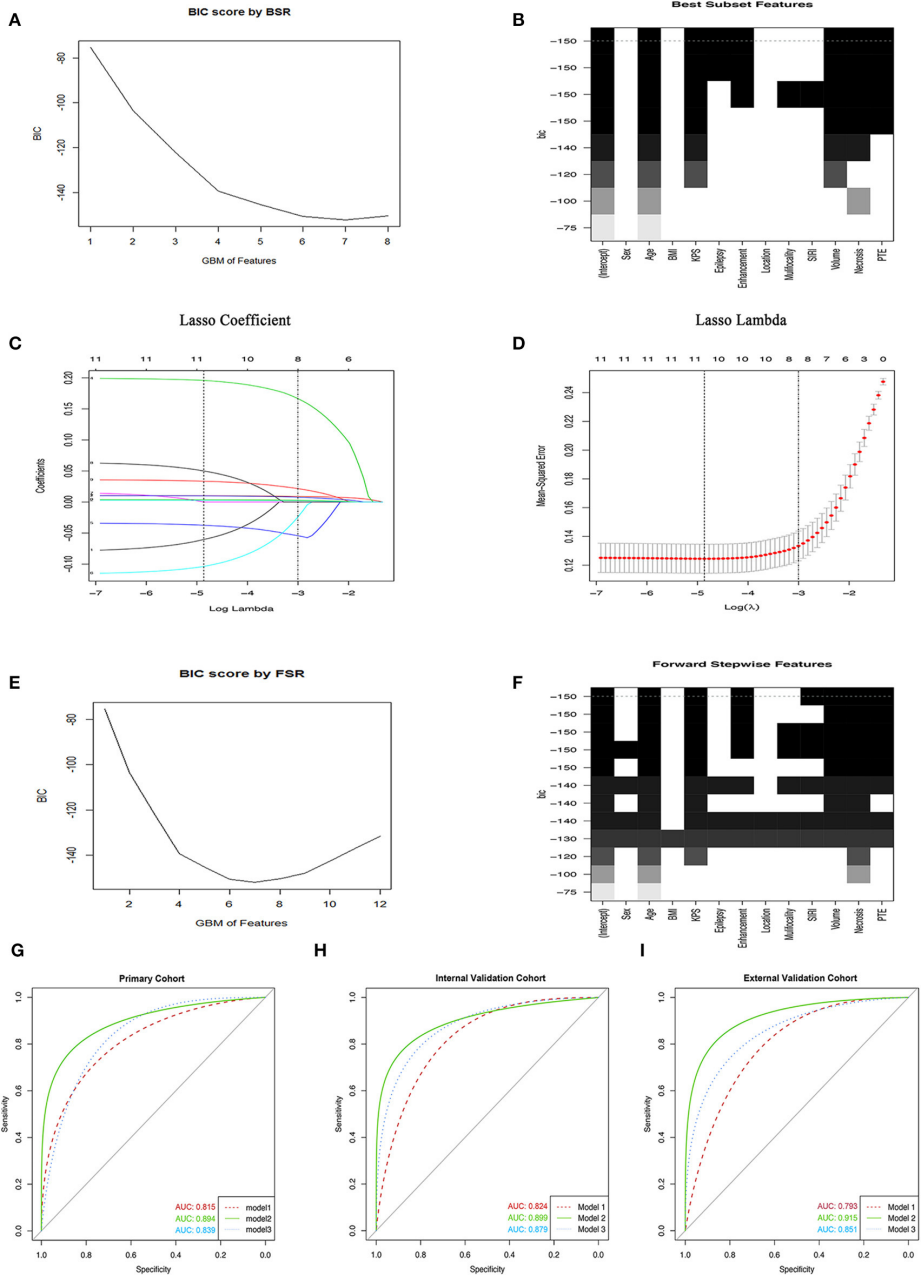
Variables selection methods. **(A,B)** The selection of variables using the BSR method. **(C)** The LASSO coefficient profile of 12 GBM-related variables in primary cohort. **(D)** 10-fold cross-validation (CV) for tuning parameter (λ) selection. **(E,F)** The FSR method was used to select variables. **(G–I)** The ROC curves of GBM in primary cohort and two validation cohorts, respectively. BIC, Bayesian information criterion; GBM, glioblastoma multiforme; BSR, best subsets regression; FSR, forward stepwise regression; BMI, body mass index; SIRI, systemic inflammation response index; pEO, preoperative epilepsy occurrence; pKPS, preoperative Karnofsky performance status; PTE, peritumoral edema.

The BSR method showed great benefits on variables selection since all possible combinations of variables were calculated and the final selected combination should be optimal based on the minimum BIC. As shown in Figures 1A,B, the selection of all 12 parameters was presented and the minimum BIC was −150.The number of final variables was 7 because there was an inflection point, shown in the broken line of Figure 1A, and the dotted line referred to the final combination based on the BSR in Figure 1B, which incorporated clinical factors and MRI features in the primary cohort. All selected variables showed significantly statistical differences in all cohorts (all *P* < 0.05; Table 2, Supplementary Tables 2, 3). Consequently, model 1 was established.

**Table 2:** Risk factors for GBM in primary cohort.

	**Model 1**			**Model 2**			**Model 3**		
**Intercept and variable**	**β**	**Adjusted OR**	***P***	**β**	**Adjusted OR**	***P***	**β**	**Adjusted OR**	***P***
		**(95% CI)**			**(95% CI)**			**(95% CI)**	
Intercept	−5.40	–	–	−6.33	–	–	−7.36	–	–
Age	0.07	1.07 (1.04–1.11)	<0.01	0.07	1.07 (1.04–1.11)	<0.01	0.07	1.07 (1.04–1.11)	<0.01
pKPS	−2.47	0.09 (0.02–0.31)	<0.01	−2.18	0.11 (0.30–0.44)	<0.01	−2.22	0.11 (0.03–0.38)	<0.01
pEO	−1.87	0.15 (0.05–0.50)	<0.05	−2.01	0.13 (0.04–0.45)	<0.01	–	–	–
SIRI	–	–	–	0.48	1.62 (1.10–2.39)	<0.05	0.43	1.53 (1.06–2.22)	<0.01
Tumor volume	0.07	1.08 (1.05–1.11)	<0.01	0.07	1.08 (1.04–1.11)	<0.01	0.07	1.07 (1.04–1.10)	<0.01
Annular enhancement	1.62	5.03 (1.79–14.14)	<0.01	1.64	5.17 (1.80–14.83)	<0.01	1.39	4.02 (1.53–10.54)	<0.01
PTE	0.02	1.02 (1.01–1.03)	<0.05	0.02	1.02 (1.01–1.04)	<0.05	0.02	1.02 (1.01–1.03)	<0.05
Tumor necrosis volume	0.09	1.09 (1.03–1.17)	<0.01	0.09	1.09 (1.03–1.16)	<0.05	0.09	1.09 (1.03–1.15)	<0.01
		C_1_-index	0.114		C_2_-index	0.244		C_3_-index	0.471
Primary cohort		0.815			0.894			0.839	
Internal validation cohort		0.824			0.899			0.879	
External validation cohort		0.793			0.915			0.851	
		AIC_1_			AIC_2_			AIC_3_	
		179.63			173.71			182.51	
			IDI _(2 vs. 1)_				IDI _(2 vs. 3)_		
			<0.01			<0.05			
			11.89%				9.14%		

*pKPS, preoperative Karnofsky performance status; pEO, preoperative epilepsy occurrence; SIRI, systemic inflammation response index; P is obtained from the multivariable logistic regression; β is regression coefficient; PTE, peritumoral edema; OR, odds ratio; AIC, the Akaike information criterion; C-index, the concordance index (the area under curve in logistic regression analysis); IDI, the integrated discrimination improvement (model 2 vs. model 1, model 2 vs. model 3)*.

### Variables Selection Using the LASSO

Considering that the number of independent variables included in the regression equation should be around 10 to 15 times the number of ending events, we further adopted the LASSO to select variables. As shown in Figures 1C,D, a coefficient profile figure was produced against the ln (λ) sequence. Two dotted vertical lines were drawn at the selected value with 10-fold cross-validation, where the optimal λ was 0.048 (1 standard error of the minimum criteria) and resulted in 8 non-zero coefficients, age, pKPS, pEO, SIRI, and Tumor necrosis volume, annular enhancement, PTE and tumor volume, being selected from all variables in the primary cohort. These variables were verified to exhibit significant differences (all *P* < 0.05; Table 2, Supplementary Tables 2, 3). Consequently, model 2 was established.

### Variables Selection Using the FSR

Herein, we also used the most common variable selection method to choose combinations of potential predictors such as the FSR. Under this predominant process operation which involved a series of steps, we also obtained 7 variables and the minimum BIC was −150 because there was an inflection point in the broken line which was shown in Figures 1E,F. Similarly, all selected variables had significant statistical difference (all *P* < 0.05; Table 2, Supplementary Tables 2, 3) and model 3 was established.

### Development of Final Prediction Model

In the primary cohort, each significant variable was first evaluated by using univariate logistic regression (Supplementary Table 3). Then multivariable logistic regression analysis demonstrated that multiple variables, including age, SIRI, Tumor necrosis volume, annular enhancement, PTE, and tumor volume, were independent risk factors, while pKPS and pEO were independent protective factors (Table 2). The choice of final prediction model was determined by AIC, the ROC curve, the C-index, and the IDI, which were also used to examine efficiency of the models (Table 2 and Figures 1G–I).

**Figure 2:**
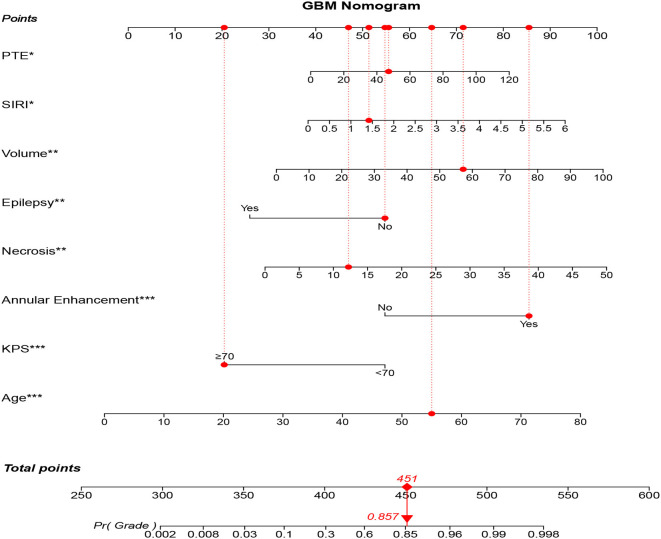
GBM-related nomogram prediction score. GBM-related nomogram was constructed to preoperatively predict GBM for glioma patients, with the age, pKPS, annular enhancement, tumor necrosis volume, pEO, tumor volume, SIRI and PTE. The nomogram showed the probability of having GBM in a randomized patient with a pathological diagnosis of GBM. SIRI, systemic inflammation response index; pEO, preoperative epilepsy occurrence; pKPS, preoperative Karnofsky performance status; PTE, peritumoral edema. *P < 0.05, **P < 0.01, ***P < 0.001.

As a result, model 2 showed the smallest AIC (173.71) among the three regression models. The discrimination of model 2 was maximum in primary cohort with the C-index 0.894 (95% CI: 0.847–0.919, *P* < 0.05). Similarly, the discrimination of model 2 was also maximum in internal validation cohort with the C-index was 0.899 (95% CI: 0.856–0.922, *P* < 0.01) and external validation cohort with C-index was 0.915 (95% CI: 0.868–0.941, *P* < 0.01). The sensitivity and specificity of model 2 were 82.8 and 79.6% in the primary cohort, 82.1 and 86.2% in the internal validation cohort, and 84.3 and 82.1% in the external validation cohort, respectively, which were more appropriate than the other models. After adding one variable to the prediction model, the IDI was significantly improved (model 2 vs. model 1:11.89%, *P* < 0.01, model 2 vs. model 3: 9.14%, *P* < 0.05). Moreover, the random forest was used to screen out candidate variables and the ROC analysis presented that the Lasso was 0.893, which preceded the random forest combination based on increase in node purity method (Supplementary Figures 2A–C and Supplementary Table 4). Therefore, the nomogram obtained from the final model was optimal (Figure 2).

### Performance of Nomogram

**Figure 3:**
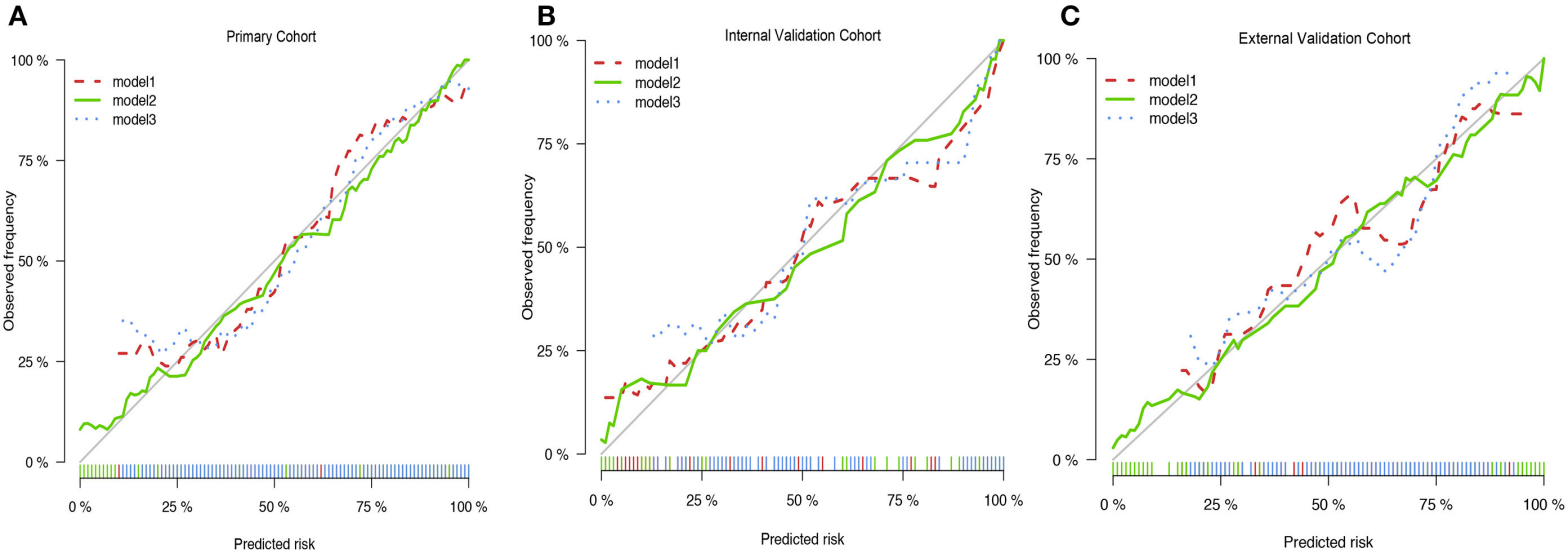
The Calibration curves of three prediction models. **(A)** The Calibration curves of three prediction models in primary cohort. **(B)** The Calibration curves of three prediction models in internal validation cohort. **(C)** The Calibration curves of three prediction models in primary cohort in external cohort.

We then verified a suitable calibration in the primary cohort and validation cohorts (Figures 3A–C). The solid straight line (the 45-degree line) showed an ideal prediction nomogram, and the other broken lines represented the observed nomogram for three models, of which a closer fit to the dashed line means a better prediction model. Consistent with the above results, the green solid broken line of calibration curves established via model 2 also showed a superior performance among the three cohorts. Moreover, the Hosmer–Lemeshow test indicated the nomogram model had a satisfactory fitting (*P* = 0.752). There were no outliers for data with the *P* < 0.05. All predictors had no multicollinearity because the VIF in all them was <1.5. The Box-Tidwell test showed a linear relationship between all continuous independent variables and the logit transformation value of the dependent variable (*P* = 0.474 for age, *P* = 0.421 for SIRI, *P* = 0.667 for tumor volume, *P* = 0.331 for PTE, *P* = 0.389 for tumor necrosis volume), and there were also no strong influential observations and high leverage cases as all of the Cook's distances were no more than 0.02 and all hat values were no more than 0.069 (Supplementary Figures 3A–C). Taken together, these results suggested that the nomogram was feasible.

### Clinical Usage

**Figure 4:**
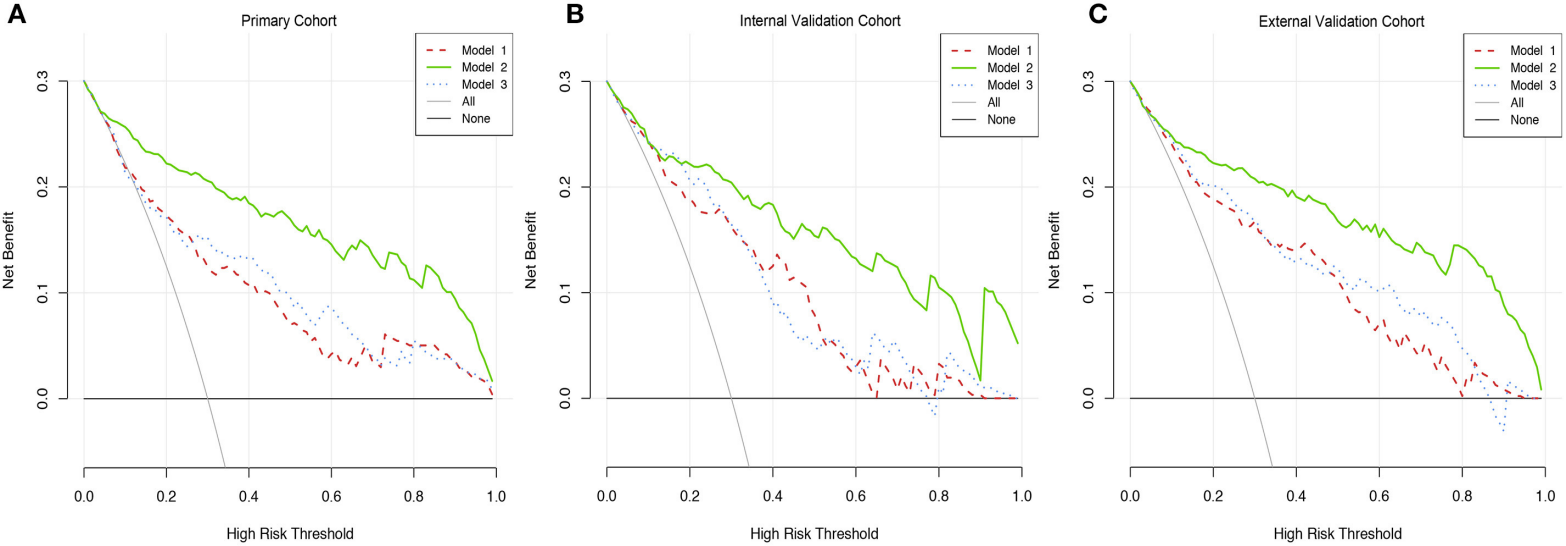
Decision curve analysis of three prediction models. **(A)** The DCA curves of three prediction models in primary cohort. **(B)** The DCA curves of three prediction models in internal validation cohort. **(C)** The DCA curves of three prediction models in primary cohort in external cohort.

Figures 4A–C showed a comparable net benefit if the threshold probability for a patient or a doctor was within a range from 0 to 0.85, according to DCA. The y-axis showed the net benefit, which was a difference value between the proportion of false positive patients and the proportion of true positive patients, weighted by the relative harm of deserting therapies compared with the negative effects of unnecessary therapies (39). The oblique smooth solid line represented a kind of hypothesis that all patients have GBM. The horizontal smooth solid line represented a kind of hypothesis that all patients have no GBM. The oblique broken lines represented all patients who were considered as GBM according to the constructed prediction model. In our current study, the decision curves in three cohorts showed that if the threshold probability was between 0 and 0.80, then using the comprehensive nomogram to preoperatively predict GBM added more benefit than treating either all or no patients, while the perfect model was the model with the highest net benefit under any threshold probability. The results also indicated that nomogram could improve current treatment standards for glioma.

## Discussion

In order to rationally promote the individual multidisciplinary integrated treatment of gliomas, the development of a more efficient and comprehensive prediction system for gliomas becomes an urgent need. Instead of adopting only a single indicator to predict glioma grade (17), we established and validated an integrated nomogram to accurately predict the probability of pathological grade in glioma patients before undergoing craniotomy. Among the 365 patients, 259 were included in a primary cohort for creating the model and the remaining cases were arranged in an internal validation cohort for validating the model in a chronological order. Additionally, we also collected 159 patients to establish an external validation cohort in other time periods to ensure the extensibility and accuracy of the prediction model. Herein, we proposed that the three cohorts were homogeneous and comparable according to the statistical analysis. Considering some limitations from using traditional statistical strategy, we adopted three advanced statistical methods to select variables in the primary cohort. As a result, these methods reduced bias generated by “data snooping” and ensured the reliability of the model.

Based on the LASSO method, we screened out eight candidate indicators to construct model 2. The performance of model 2 was the most appropriate among the three models with the criterions. The LASSO, which not only avoided the mismatch between the number of independent variables and the ending events but also exceeded the high collinearity of the selected variables, has been proven to perform more favorably in a dataset with a lower number of ending events compared to the BSR and the FSR (40). Additionally, the ROC and calibration analysis were performed to validate the discrimination and calibration of model 2 among the three cohorts. With this combination, patients with suspicious GBM could be precisely identified before the surgical resection, which provided more evidence to the surgeon to formulate the surgical strategy. The rationality and feasibility of the data was also verified for further confirmation of the models. Moreover, the ability of discrimination between the Lasso and random forest methods were compared and the result suggested that the combination of selected variables based on increased mean squared error in random forest was similar to the Lasso while the other combination exhibited significant deficiency in random forest compared to Lasso according to the ROC analysis (Supplementary Figures 2A–C and Supplementary Table 4). Eventually, an integrated nomogram for pre-surgical prediction of GBM was established based on these novel findings and was confirmed by DCA analysis. Altogether, our nomogram could be helpful to separate GBM patients via a non-invasive method before surgery and make appropriate clinical therapeutic decisions.

To gain more insight into the clinical relevance of our nomogram in gliomas, the correlation between the nomogram score and overall survival rate were investigated based on the different WHO grades of gliomas. The results showed that the high nomogram score derived from our methods was strongly correlated with all WHO grades and overall survival of glioma patients. Additionally, the time-dependent ROC analysis indicated that the nomogram was appropriate (Supplementary Figures 4A,B). Moreover, we divided all glioma samples into two groups based on their malignant characters, GBM (Grade IV) and lower-grade gliomas (Grade II and III), according to the TCGA standards. Moreover, the Kaplan–Meier analysis showed that an elevated nomogram score revealed more severe prognosis in either LGG or GBM, indicating that our model was sensitive to gliomas despite the WHO grade (Supplementary Figures 4C,D). The pKPS and pEO could be independent protective factors while age, SIRI, PTE, annular enhancement, tumor volume, and tumor necrosis volume could be independent risk factors (Supplementary Figure 5). Lastly, the correlation between nomogram score and survival in patients who received different post-surgical treatment were explored. The results showed that increased nomogram score could be correlated with poor prognosis despite the patient having received TMZ/radiation or not (Supplementary Figures 6A–D). Altogether, these data suggested that our model could be used as a predictor for tumor grade and revealed prognosis in gliomas as well; moreover, it is independent from eventual modification of survival caused by post-surgical treatment.

Multiple studies that aimed to develop prognostic markers for malignant tumors by combining clinical characteristics with preoperative examinations have been previously reported (41, 42). It has been proven that the nomogram based on multimodal biomarkers could successfully predict axillary lymph node (ALN) metastasis in patients with breast cancer before surgery, which was rational in the training cohort (C-index: 0.856) and reliable in the validation cohort (C-index: 0.841) (43). Although a previous study also established a non-invasive risk score to predict ALN metastasis, the C-index remained at only 0.74 and showed a lack of comprehensive index (44). Therefore, integrated profiling could provide a more accurate preoperative diagnosis and result in reasonable clinical decisions.

Recent studies have indicated that MRI features of glioma, including tumor necrosis, volume, enhancement, and peritumoral edema, could present abundant information about glioma heterogeneity (26, 45, 46). Henker et al. (26) also demonstrated that preoperatively measured necrosis volume and necrosis-tumor ratios are the most important radiological features of GBM with a strong influence on OS. Liu et al. (45) investigated the correlation between progression-free survival (PFS) and MRI features among 300 patients with LGG (216 cases in a training cohort and 84 cases in a validation cohort). The results showed that MRI features were significantly associated with PFS (*P* < 0.05) and a comprehensive nomogram which had favorable discrimination and calibration for prediction of PFS was established; moreover, researchers considered that increased risk score of nomogram implied malignancy of glioma. Meanwhile, Muccio et al. (46) analyzed the differentiation between cerebral metastases (CM) and GBM for MRI and they suggested that the signal alteration in the adjacent cortex was specific for GBM and peripheral rim sign was specific for CM. Herein, we speculated that MRI reveals malignant characteristics of gliomas, including proliferation, tumor invasion, and microenvironment changes (47, 48). To this end, tumor volume, necrosis volume, and peritumoral edema volume were extracted from images from glioma patients and then enrolled in our nomogram. Compared to an isolated predictor of SIRI, the ROC and calibration curves of our nomogram were significantly improved (Supplementary Figures 7A,B).

Tumor-related inflammation has long been considered as an important hallmark of cancer (49). Accumulating evidence suggested that tumorigenesis and invasion of the tumor were closely related to the chronic non-specific inflammation process and were thought to play a crucial role in the survival of patients. This progress was mediated by inflammation cells and cytokines that can present and be detected in peripheral blood (50). Wang et al. (17) found that nutrition-related markers, including albumin-to-globulin and prognostic nutrition index (PNI), were negatively associated with glioma grades and were remarkably reduced in GBM in contrast to LGG (all *P* < 0.01). The diagnostic value of hematological markers in predicting glioma grade combined with age and PNI showed fantastic discrimination with AUC of 0.750. Furthermore, Geng et al. (51) found that the median overall survival rate in patients with SIRI≤1.2 was significantly higher than in patients with SIRI>1.2 and the nomogram including SIRI could more accurately predict OS compared with the TNM staging system. Therefore, an inflammation index such as SIRI proved to be a potential index for predicting tumorigenesis and metastasis of glioma and should be involved in the prediction model. In our study, SIRI was measured by counting of PBIC and it was found that SIRI was positively correlated with the pathological grade of gliomas (*P* < 0.01, Supplementary Table 2). Taken together, we constructed and evaluated a comprehensive probabilistic score for the preoperative prediction of GBM in glioma, which could be a crucial method for early diagnosis and support more rational treatment of GBM.

Although our nomogram showed encouraging discrimination and calibration among the three cohorts, there were still some limitations. Since a retrospective method was used in this study, inherent deviations such as selection deviation and detection deviation were inevitably generated. Also, continuous monitoring for the variation of some parameters cannot be achieved. Further, molecular mechanism studies and large-scale and multi-center clinical trials needed to be performed to modify the model.

## Conclusion

In conclusion, our study showed a novel preoperative model incorporated clinically relevant variables and imaging features with laboratory data that could be used for preoperative prediction in glioma patients, thus providing more reliable evidence for surgical decision-making.

## Data Availability Statement

The data generated during this study are included in this article. Raw data are available upon reasonable request.

## Ethics Statement

This study was approved by the Ethics Committee, the First Affiliated Hospital of Xi'an Jiaotong University. This is a retrospective study, for which formal consent is not required.

## Author Contributions

WW: collection and/or assembly of data, data analysis and interpretation, manuscript writing, methodology, and software. ZD: collection and/or assembly of data, data analysis and interpretation, manuscript writing, and editing. WA: data analysis, manuscript writing, and interpretation. YW: manuscript writing and project administration. JX: manuscript writing and project administration. LZ: manuscript writing. BL: conception/design. MW: conception/design, supervision, and editing. JW: conception/design, supervision, and editing. All authors contributed to the article and approved the submitted version.

## Conflict of Interest

The authors declare that the research was conducted in the absence of any commercial or financial relationships that could be construed as a potential conflict of interest.
