# Supplementary material for: Preoperative Prediction Nomogram Based on Integrated Profiling for Glioblastoma Multiforme in Glioma Patients
Source: Front Oncol. 2020 Oct 21;10:1750. doi: 10.3389/fonc.2020.01750 (PMC7609958; doi:10.3389/fonc.2020.01750)
Supplement: Supplementary file 1 [file Data_Sheet_1.pdf]

Supplementary Material

1 Supplementary Figures and Tables

1.1 Supplementary Figures

Supplementary Figure 1. The flow diagram for this study.

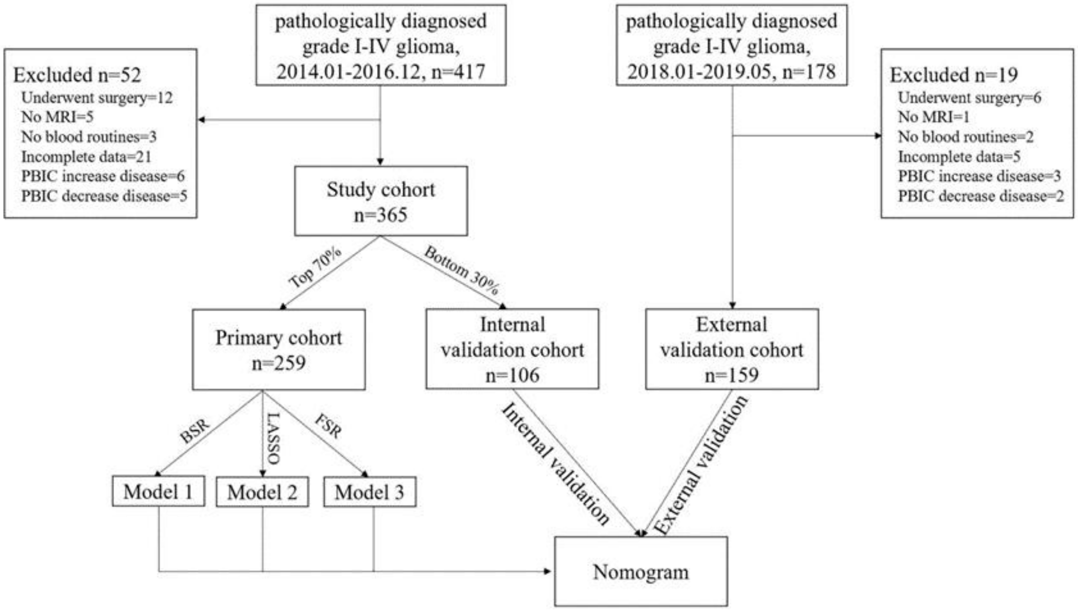

Abbreviations: PBIC, peripheral blood inflammatory cell; BSR, the best subsets regression; LASSO, the least absolute shrinkage and selection operator; FSR, the forward stepwise regression.

Supplementary Figure 2. A, the random forest variable importance measures. B, the selection variable by 10 times cross-validation. C, the ROC analysis for different method. Especially, the combination of selected variables based on increase in mean squared error in random forest was the same as the Lasso and the other combination for random forest was worse than Lasso according to ROC analysis.

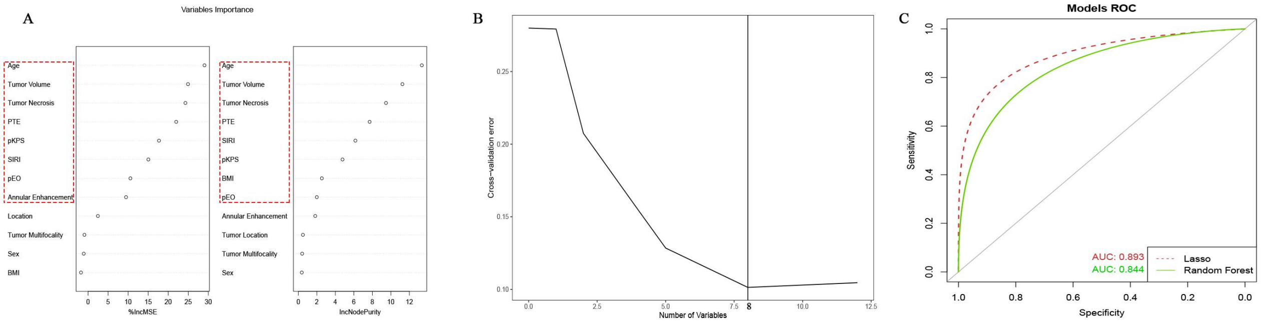

Abbreviations: BMI, body mass index; pKPS, preoperative Karnofsky performance status; pEO, preoperative epilepsy occurrence; SIRI, systemic inflammation response index; PTE, peritumoral edema; %IncMSE: increase in mean squared error; IncNodePurity: increase in node purity.

Supplementary Figure 3. A, The linear relationship between all continuous independent variables and the logit transformation value of the dependent variable. The Box-Tidwell test showed *P* was 0.474 for age, *P* was 0.421 for SIRI, *P* was 0.667 for tumor volume, *P* was 0.331 for PTE and *P* was 0.389 for Tumor necrosis volume, respectively. B, The Cook's distance for all observations. The Cook's distance was no more than 0.02 which was calculated by $\frac{4}{n-p-1}$. C, The hat value for all observations. The two horizontal smooth dotted lines represented 2 and 3 times for the mean of hat value which was calculated using $\frac{p}{n}$. There were no high leverage cases due to all hat values were no more than 0.069. Especially, n referred to sample capacity and p referred to the number of independent variables.

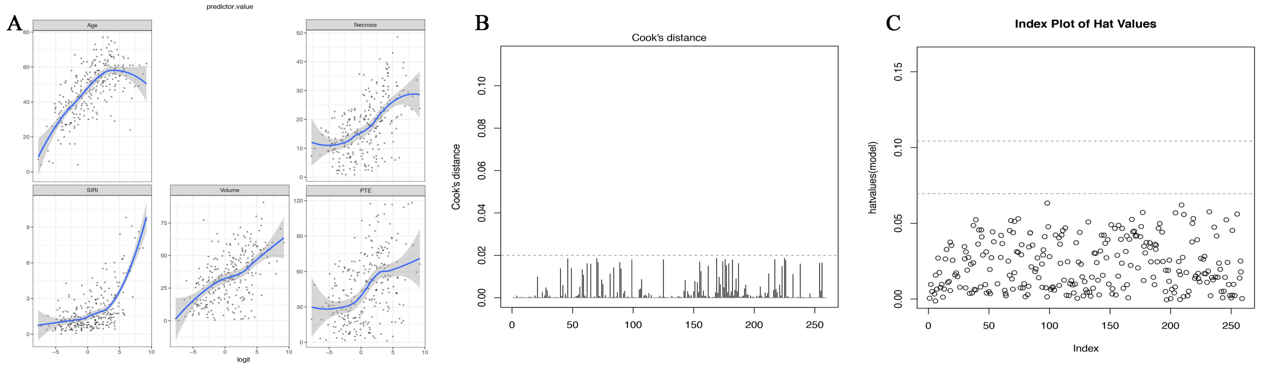

Abbreviations: SIRI, systemic inflammation response index; PTE, peritumoral edema

Supplementary Figure 4. A, The Kaplan-Meier analysis for nomogram score in all grade gliomas (P=0.0015, with log-rank test). B, The time-dependent ROC analysis for all glioma patiens. C, The Kaplan-Meier analysis for nomogram score in lower grade gliomas (P=0.013, with log-rank test). D,

The Kaplan-Meier analysis for nomogram score in glioblastoma multiforme (P=0.034, with log-rank test). Especially, the cut off values were calculated by X-tile.

A

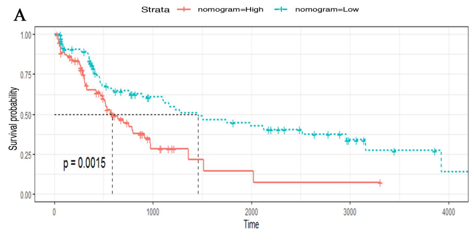

B

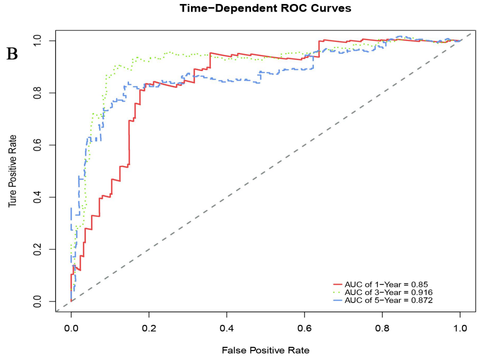

All Grade Gliomas

cut off=89

C

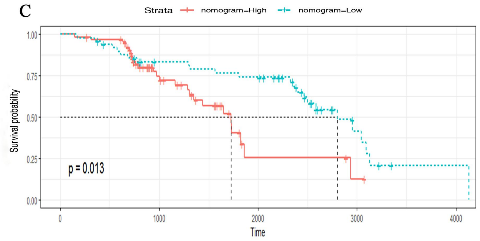

D

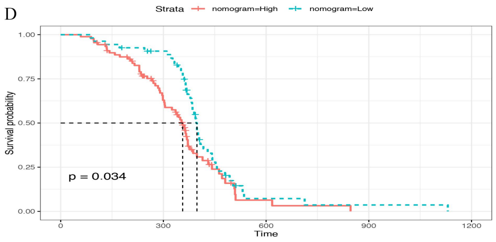

Lower Grade Gliomas

cut off=147

Glioblastoma Multiforme

cut off=217

Supplementary Figure 5. The forest plot of multivariable Cox regression.

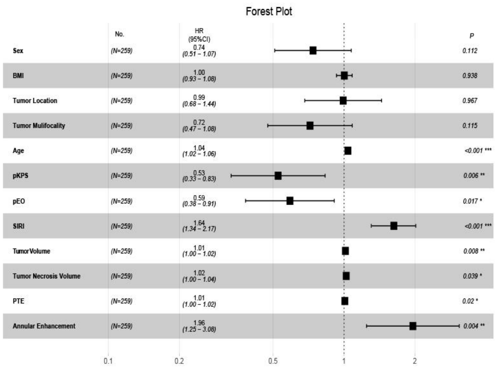

Abbreviations: BMI, body mass index; pKPS, preoperative Karnofsky performance status; pEO, preoperative epilepsy occurrence; SIRI, systemic inflammation response index; PTE, peritumoral edema.

Supplementary Figure 6. A, The Kaplan-Meier analysis of nomogram score in surgery plus radiation treatment group (*P*=0.041, with log-rank test). B, The Kaplan-Meier analysis of nomogram score in surgery plus TMZ treatment group (*P*=0.016, with log-rank test). C, The Kaplan-Meier analysis of nomogram score for LGG in surgery plus radiation and TMZ treatment group (*P*=0.002,

with log-rank test). D, The Kaplan-Meier analysis of nomogram score for GBM in surgery plus radiation and TMZ treatment group (*P*=0.041, with log-rank test). Especially, the number of GBM patients was too few to analyze in in surgery plus radiation treatment group (No.=3) and in surgery plus TMZ treatment group (No.=7). The cut off values were calculated by X-tile.

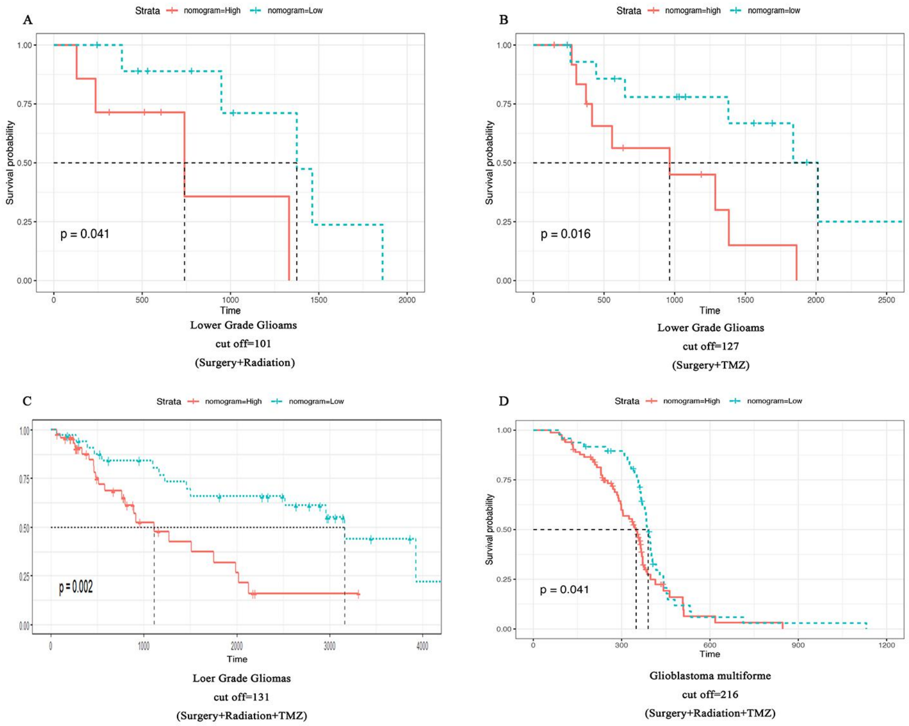

Supplementary Figure 7. A, The ROC analysis for nomogram and single predictor in primary cohort. B, The Calibration curves analysis for nomogram and single predictor in primary cohort.

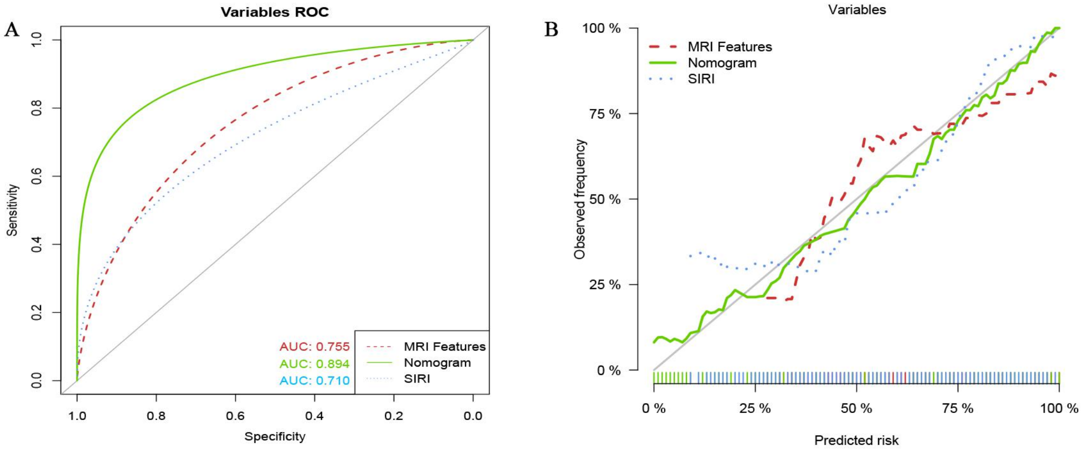

Abbreviations: SIRI, systemic inflammation response index.

1.2 Supplementary Tables

Supplementary Table 1. Clinical Characteristics of Patients in the Primary and Validation Cohorts

Characteristics	LGG			*P*	GBM			*P*
	Primary Cohort	Internal Validation Cohort	External Validation Cohort		Primary Cohort	Internal Validation Cohort	External Validation Cohort	
Sex				0.713				0.547
Male	60 (51.7%)	27 (56.3%)	38 (48.7%)		79 (55.2%)	31 (53.4%)	50 (61.7%)	
Female	56 (48.3%)	21 (43.8%)	40 (51.3%)		64 (44.8&)	27 (46.6%)	31 (38.3%)	
Age, median (IQR), years	37.50 (28.25-47.75)	39.00 (31.50-50.50)	39.00 (28.75-47.25)	0.701	55.00 (49.00-61.00)	56.00 (47.00-63.25)	57.00 (53.00-62.00)	0.373
BMI, median (IQR), kg/m^2	21.58 (19.97-23.42)	22.36 (21.24-23.77)	22.66 (21.30-23.69)	0.646	22.01 (20.96-23.68)	21.92 (20.65-23.97)	22.48 (21.11-23.99)	0.713
pKPS				0.436				0.947
<70	5 (4.3%)	1 (2.1%)	1 (1.3%)		73 (51.0%)	28 (48.3%)	40 (49.4%)	
≥70	111 (95.7%)	47 (97.9%)	77 (98.7%)		70 (49.0%)	30 (51.7%)	41 (50.6%)	
pEO				0.993				0.255
Yes	60 (51.7%)	25 (52.1%)	41 (52.6%)		28 (19.6%)	11 (19.0%)	23 (28.4%)	
No	56 (48.3%)	23 (47.9%)	37 (47.4%)		115 (80.4%)	47 (81.0%)	58 (71.6%)	
SIRI, median (IQR)	0.92 (0.56-1.50)	0.89 (0.54-1.31)	0.76 (0.52-1.19)	0.297	1.80 (1.18-2.36)	1.33 (1.05-2.22)	1.63 (1.07-2.57)	0.311
Tumor volume, median (IQR), cm^3	23.22 (11.60-31.17)	21.78 (12.15-32.01)	22.44 (12.65-31.96)	0.142	47.89 (28.48-59.31)	46.12 (27.66-58.89)	47.34 (28.17-58.92)	0.224
Tumor location				0.337				0.431
Supratentorial	64 (55.2%)	26 (54.2%)	41 (52.6%)		74 (51.7%)	31 (53.4%)	44 (54.3%)	
Infratentorial	52 (44.8%)	22 (45.8%)	37 (47.4%)		69 (48.3%)	27 (46.6%)	37 (45.7%)	

Tumor multifocality				0.823				0.469
Yes	6 (5.2%)	3 (6.3%)	3 (3.8%)		44 (30.8%)	23 (39.7%)	26 (32.1%)	
No	110 (94.8%)	45 (93.8%)	75 (96.2%)		99 (69.2%)	35 (60.3%)	55 (67.9%)	
Annular enhancement				0.947				0.803
Yes	56 (48.3%)	22 (45.8%)	38 (48.7%)		119 (83.2%)	46 (79.3%)	66 (81.5%)	
No	60 (51.7%)	26 (54.2%)	40 (51.3%)		24 (16.8%)	12 (20.7%)	15 (18.5%)	
Tumor necrosis volume, median (IQR), cm^3	12.75 (8.18-18.06)	11.48 (8.53-17.79)	12.11 (8.07-18.37)	0.198	20.65 (12.25-29.20)	19.88 (12.04-29.16)	20.77 (13.03-29.98)	0.379
PTE, median (IQR), cm^3	31.08 (18.03-47.97)	30.61 (18.28-48.12)	31.33 (17.91-47.72)	0.267	52.97 (24.81-76.04)	53.18 (24.78-75.61)	52.22 (23.65-76.28)	0.188

IQR, interquartile range; BMI, body mass index; pKPS, preoperative Karnofsky performance status; pEO, preoperative epilepsy occurrence; LGG, lower grade glioma; GBM, glioblastoma multiforme; SIRI, systemic inflammation response index; PTE, peritumoral edema; *P* was obtained from the Kruskal-Wallis H test and the χ^2 test.

Supplementary Table 2. Selected Variables in the Primary and Validation Cohorts

	Primary Cohort		*P*	Internal Validation Cohort		*P*	External Validation Cohort		*P*
Selected Variables	LGG	GBM		LGG	GBM		LGG	GBM	
Age, median (IQR), years	37.50 (28.25-47.75)	55.00 (49.00-61.00)	<0.01	39.00 (31.50-50.50)	56.00 (47.00-63.25)	<0.01	39.00 (28.75-47.25)	57.00 (53.00-62.00)	<0.01
pKPS			<0.01			<0.01			<0.01
<70	5 (4.3)	73 (51.0)		1 (2.1)	28 (48.3)		1 (1.3)	40 (49.4)	
≥70	111 (95.7)	70 (49.0)		47 (97.9)	30 (51.7)		77 (98.7)	41 (50.6)	
pEO			<0.01			<0.01			<0.01
Yes	60 (51.7)	28 (19.6)		25 (52.1)	11 (19.0)		41 (52.6)	23 (28.4)	
No	56 (48.3)	115 (80.4)		23 (47.9)	47 (81.0)		37 (47.4)	58 (71.6)	
SIRI, median (IQR)	0.92	1.80	<0.01	0.89	1.33	<0.01	0.76	1.63	<0.01

	(0.56-1.50)	(1.18-2.36)		(0.54-1.31)	(1.05-2.22)		(0.52-1.19)	(1.07-2.57)	
Tumor volume, median (IQR), cm^3	23.22<br>(11.60-31.17)	47.89<br>(28.48-59.31)	<0.01	21.78<br>(12.15-32.01)	46.12<br>(27.66-58.89)	<0.01	22.44<br>(12.65-31.96)	47.34<br>(28.17-58.92)	<0.01
Tumor necrosis volume, median (IQR), cm^3	12.75<br>(8.18-18.06)	20.65<br>(12.25-29.20)	<0.05	11.48<br>(8.53-17.79)	19.88<br>(12.04-29.16)	<0.05	12.11<br>(8.07-18.37)	20.77<br>(13.03-29.98)	<0.05
Annular enhancement			<0.01			<0.01			<0.01
Yes	56 (48.3%)	119 (83.2%)		22 (45.8%)	46 (79.3%)		38 (48.7%)	66 (81.5%)	
No	60 (51.7%)	24 (16.8%)		26 (54.2%)	12 (20.7%)		40 (51.3%)	15 (18.5%)	
PTE, median (IQR), cm^3	31.08<br>(18.03-47.97)	52.97<br>(24.81-76.04)	<0.01	30.61<br>(18.28-48.12)	53.18<br>(24.78-75.61)	<0.01	31.33<br>(17.91-47.72)	52.22<br>(23.65-76.28)	<0.01

LGG, lower grade glioma; GBM, glioblastoma multiforme; IQR, interquartile range; pKPS, preoperative Karnofsky performance status; pEO, preoperative epilepsy occurrence; SIRI, systemic inflammation response index; PTE, peritumoral edema; *P* was obtained from the Kruskal-Wallis H test and the χ^2 test.

Supplementary Table 3. Risk Factors for GBM in Primary Cohort

Variable	Models		
	β	OR（95% CI）	*P*
Age	0.99	1.10<br>(1.08-1.13)	<0.01
pKPS	-3.01	0.05<br>(0.02-0.12)	<0.01
pEO	-1.19	0.30<br>(0.16-0.58)	<0.01
SIRI	0.73	2.08<br>(1.60-2.71)	<0.01
Tumor volume	0.07	1.07<br>(1.05-1.09)	<0.01

Annular enhancement	1.67	5.31<br>(3.00-9.39)	<0.01
PTE	0.03	1.03<br>(1.02-1.04)	<0.01
Tumor necrosis volume	0.10	1.11<br>(1.07-1.15)	<0.01

pKPS, preoperative Karnofsky performance status; pEO, preoperative epilepsy occurrence; SIRI, systemic inflammation response index; PTE, peritumoral edema; *P* was obtained from the univariable logistic regression; β was regression coefficient; OR, odds ratio.

Supplementary Table 4. The random forest variable importance measures

variable	%IncMSE	IncNodePurity
Age	0.058267	13.62311
Tumor Necrosis Volume	0.051204	10.04949
Tumor Volume	0.045594	10.96671
PTE	0.033531	7.658929
pKPS	0.023878	4.157103
SIRI	0.019902	6.140776
pEO	0.00989	2.13419
Annular Enhancement	0.007508	1.700399
Tumor Multifocality	0.000538	0.383942
Tumor Location	0.000335	0.486894
Sex	0.000173	0.335287
BMI	-0.00174	2.727991

Abbreviations: BMI, body mass index; pKPS, preoperative Karnofsky performance status; pEO, preoperative epilepsy occurrence; SIRI, systemic inflammation response index; PTE, peritumoral edema; %IncMSE: increase in mean squared error; IncNodePurity: increase in node purity.

1.3 Supplementary materials and methods

The peritumoral edema, volume and cystic necrosis of tumors were calculated by the following formulas [25]:

- cuboid: $V = a \times b \times c$ (total axes measured)
- (tri-axial) ellipsoid: $V = \frac{4}{3} \times \pi \times a \times b \times c$ (semi-axes measured; a > b> c)

  equivalent to $V = \pi \times a \times b \times c \div 6$
- oblate spheroid: $V = \frac{4}{3} \times \pi \times a^2 \times b$ (semi-axes measured; a = b > c) or
- prolate spheroid: $V = \frac{4}{3} \times \pi \times a \times b^2$ (semi-axes measured; a = b < c)
- sphere: $V = \frac{4}{3} \times \pi \times r^3$ (semi-axes measured; a = b = c)
